# Conjugated Conductive Polymer Materials and its Applications: A Mini-Review

**DOI:** 10.3389/fchem.2021.732132

**Published:** 2021-09-06

**Authors:** Huizhi Lu, Xunlai Li, Qingquan Lei

**Affiliations:** ^1^ College of Economics and Management, Qingdao University of Science and Technology, Qingdao, China; ^2^ Institute of Advanced Electrical Materials, Qingdao University of Science and Technology, Qingdao, China

**Keywords:** conjugated polymers, organic field-effect transistors, sensors, organic solar cells, coating

## Abstract

Since their discovery 50 years ago, conjugated conducting polymers have received increasing attention owing to their unique conductive properties and potential applications in energy storage, sensors, coatings, and electronic devices such as organic field-effect transistors, photovoltaic cells, and light-emitting devices. Recently, these materials have played a key role in providing a more comfortable environment for humans. Consequently, the development of novel, high-performance conjugated conductive materials is crucial. In this mini-review, the progress of conjugated conductive materials in various applications and the relationship between the chemical structures and their performances is reviewed. This can aid in the molecular design and development of novel high-performance conjugated polymer materials.

## Introduction

Conjugated conducting polymers can be divided into the following three categories according to their structural characteristics and conducting mechanism: electron conducting polymers, ionic conducting polymer, and redox polymer. Among these, the carriers of electron conducting polymers are free electrons and their common characteristic is a long π-conjugated system in the molecular skeleton, which results in delocalised electrons. Thus, these molecules are called conjugated conducting polymers. To efficiently increase the movement of electrons in the π-system, the energy level difference caused by energy band splitting should be reduced to easily overcome the energy difference between the full and empty bands. The ‘doping’ method can be used to change the distribution state of electrons in the energy band because conjugated polymers are easily oxidised or reduced. Therefore, almost 50 years ago, scientists found that the conductivity of polyacetylene can be improved by more than 10 orders *via* chemical doping, which was the first report on conjugated conducting polymers with conductive properties (Shirakawa et al., 1977). Recently, with the gradual development and understanding of the conjugated conductive polymer mechanism, conjugated conducting polymers have become a popular topic. They are widely used in organic solar cells (OSCs), organic field effect transistors (OFETs), sensors, and colour-changing coatings, due to advantages including light weight, low cost, good stability, and excellent optical and electrical properties (Zhang et al., 2019a; Bao et al., 2020; Deng et al., 2020; Zhang et al., 2020; Dai et al., 2021). Recently, conjugated conductive polymers have played a key role in improving our standard of living. The development of novel, high-performance conjugated conductive materials as well as their applications is crucial. Despite the rapid development of conjugated conducting polymers, their application has rarely been reviewed (Guo et al., 2014; Yi et al., 2015; Deng et al., 2019). In this mini-review, the research progress of conjugated conducting polymers in OSCs, OFETs, sensors, coatings, and other applications, as well as the relationship between the chemical structures and their performances, is reviewed. Furthermore, this review provides not only a reference and prospect for future applications, but also the development prospects of ideal conjugated conductive materials.

## Organic Solar Cell

The core of organic solar cells is the use of photosensitive organic materials as semiconductor materials which can generate voltage and current to achieve solar power generation *via* the photovoltaic effect. Recently, organic solar cell-based organic conjugated polymers, which are the leading third-generation low-cost photovoltaic technology, have attracted increasing attention due to their easy processing and low cost (Chen et al., 2016; Liu et al., 2017; Li et al., 2020). Till now, the organic solar cells exhibited efficiency over 17% has reported, which is compete-able with the commercial silicon based solar cells (Zhan et al., 2020).

**FIGURE 1:**
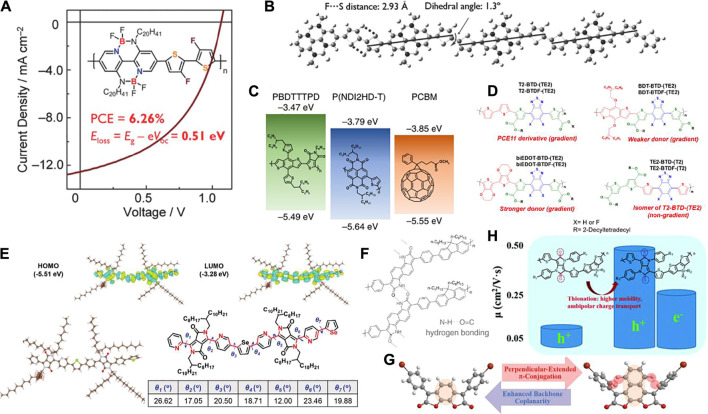
**(A)** Chemical structure of the polymer as well as the J–V curves of OSC device **(B)** Optimised configuration of the model compound of P-BNBP-fBT, **(C)** Chemical structures and energy levels of PBDTTTPD, PCBM, and P(NDI2HD-T), **(D)** Chemical structures of the eight polymers, **(E)** DFT calculations for polymer dimer with full side chains, **(F)** Hydrogen-bonded naphthodipyrrolidione-based polymer, **(G)** Chemical structures of 1,8-benzodifuranone and 1,10-naphthodifuranone, **(H)** Chemical structures of the two polymers as well as their charge transfer mobility.

**FIGURE 2:**
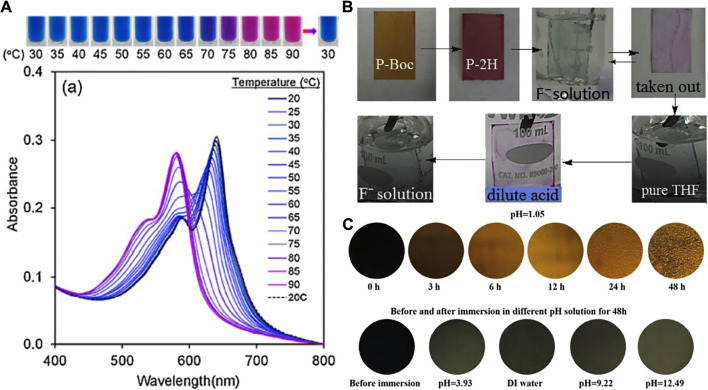
**(A)** Absorption spectra of PDA(8, 9)/PVP10 nanocomposites measured upon increasing temperature. Dashed lines represent spectra measured upon the consecutive cooling (photographs of the colour transition are illustrated above the spectra), **(B)** Photos of dried P-2H film in THF solution of fluoride anion or HCl with solute concentration of 10^–5^ M **(C)** Colour change in the 1,4-BDF-based epoxy coating samples after immersion in different pH solutions and digital images of the coating samples after immersion in the other pH solutions (from pH = 3.93 to pH = 12.49).

For the conjugated polymer in OSC applications, the materials should not only have broad optical absorption matching well with the solar energy spectrum, but also have suitable energy levels as well as good charge transfer mobility. Thus, the molecular design is crucial. In 2016, Long et al. reported a polymer, P-BNBP-fBT, based on alternating copolymerisation with double B←N bridged bipyridine (BNBP) and 3, 3′-difluoro-2, 2′-bithiophene (fBT) by the Schiff-base formation reaction, which exhibited a power conversion efficiency (PCE) of up to 6.26% at a photon energy loss (E_loss_) of only 0.51 eV (Figure 1A) (Long et al., 2016). The high PCE could be attributed to: 1) the polymer has a good planar structure because its planar conformation can be ‘locked’ by the F…S interaction, which has been confirmed by the B3LYP/6–31G* level of theory (Figure 1B); 2) a large number of F atoms in the easily modified main chain of the polymer helps to deepen the LUMO energy level and N atoms are conducive to the formation of intermolecular hydrogen bonds to improve the stability of the molecule. The polymer backbone configuration and the ‘locked’ coplanar conformation of the fBT unit endow P-BNBP-fBT with good crystallinity and high electron mobility as an excellent donor in OSCs. This work demonstrates the potential of conjugated conducting polymers in OSCs, such that advances in all-polymer solar cell (all-PSC) device performance can be achieved.

The development of solar cells should include improved efficiency, while simultaneously overcoming the difficulties in practical applications that are ubiquitous in daily life. Theoretical performance and practical application should be considered. Kim et al. developed highly efficient and mechanically robust all-PSCs using poly[4, 8-bis(5-(2-ethylhexyl) thiophen-2-yl)benzo[1, 2-b:4, 5-b’]dithiophene-alt-1, 3-bis(thiophen-2-yl)-5-(2-hexyldecyl)-4H-thieno[3,4-c]pyrrole-4, 6 (5H)-dione] (PBDTTTPD) as the electron donor and poly[[N,N′-bis(2-hexyldecyl)-naphthalene-1, 4, 5, 8-bis(dicarboximide)-2, 6-diyl]-alt-5, 5′-thiophene] (P(NDI2HD-T)) as the electron acceptor, which achieved a high PCE of 6.64%, higher than that of control fullerene PSCs (PCE = 6.12%) (Figure 1C) (Kim et al., 2016). The enhanced performance of all-PSCs is mainly attributed to the high V_OC_ (1.06 V) due to the better alignment of highest occupied molecular orbital (HOMO) and lowest unoccupied molecular orbital (LUMO) energy levels. In addition, many S atoms on PBDTTTPD enhance the electron-donating ability of the molecule, and the large conjugated structure and good molecular planarity provide conditions for high carrier mobility and PCE. This work not only shows the high performance of conjugated conductive polymers in OSCs, but also provides strong operability for extensive use in daily life.

Recently, research has focused on non-fullerene acceptors (NFAs) in OSCs because of their advantages, including greater optical, electrochemical, and structural flexibility compared to their fullerene counterparts (Lin et al., 2015; Nielsen et al., 2015; Hou et al., 2018; Zhang et al., 2018a). In 2019, Reynolds’s et al. reported a series of polymers by minimal structure modification with varying electron donors (Figure 1D) (Jones et al., 2019). These polymers are consisted of a fluorinated/non-fluorinated benzothiadiazole strong acceptor moiety, a thiophene ester weak acceptor, and various donor units composed of bithiophene, biEDOT, and benzodithiophene to form six acceptor gradient and two non-gradient polymers. The results showed that optimised NFA solar cells based on ITIC-4F paired with each of the top-performing polymers produced an average PCE of up to 7.3% for TE2-BTDF-(T2) (non-gradient) and 3.6% for T2-BTDF-(TE2) (gradient). The differences might be due to the formation of an intramolecular charge transfer that occurred for the acceptor gradient. This work stresses the importance of acquiring accurate ionisation energies and electron affinities when characterising solar cell energetics, since differences as small as 0.1 eV in the offsets can make a significant impact on overall charge collection.

## Organic Field Effect Transistor

Organic field-effect transistors (FETs) are indispensable electronic devices based on organic materials, which use electric fields to control the conductivity of materials. Research on conjugated conductive polymers has become an important aspect in OFETs owing to advantages including flexibility, easy processing, rich variety, and low cost. Further breakthroughs in the business and industry of OFETs is expected (Zou et al., 2021). However, the low carrier mobility of these conjugated polymers compared to the silicon-based field-effect transistors is a major disadvantage which needs to be addressed.

There are three factors that affect the high-performance OFET: charge transfer mobility, threshold voltage, and current on/off ratio. Among these, the charge carrier mobility is one of the most important and challenging, typically for high electron transfer mobility. The polymer molecular structure and device fabrication techniques are essential to obtain high-performance OFETs. In 2020, Liu et al. developed a new method to improve carrier mobility in OFETs using the synergistic effect of pyridine and selenophene in the backbone of a DPP-based copolymer (Figure 1E) (Liu et al., 2020). The study found that the pyridine DPP- and selenophene-based copolymer (PDPPy-Se) exhibited low LUMO and HOMO energy levels due to molecular packing and ordering, which increased the hole transfer barrier and decreased the electron transfer barrier. In addition, the study concluded that the annealing process increases the formation of microcrystals and improves π-stacking and lamellar stacking, which is beneficial to carrier migration. This work demonstrated that the synergistic use of pyridine and selenophene in the backbone of a diketopyrrolopyrrole-based copolymer is a favourable energy level, typically to reduce the LUMO energy level, for electron injection and relatively ordered molecular packing, which is a feasible method to improve carrier mobility and plays a key role in the development of n-type bipolar organic transistors. It also highlights the promising future of conjugated conductive polymers in OFETs.

In 2018, Zhang et al. (2017) reported that introducing hydrogen bonding between the amide and carbonyl into the polymer not only result in polymer self-assembly, but also increases the electron mobility by a factor of 40 compared to its precursor polymer (Figure 1F). Recently, Deng et al. (2018) introduced S and F atoms to adjust the polymer backbone and obtained a polymer with hole mobility up to 0.65 cm^2^ V^−1^ s^−1^. Zhang et al. then reported that the main chain coplanarity of polymer semiconductors is more essential than the sole extension of π-conjugation (especially perpendicularly to polymer main chains) after investigating two benzo/naphtodifuranone-based polymers (Figure 1G) (Li et al., 2021). The same group also found that substituting the oxygen atoms for sulfur atoms in the diketopyrrolopyrrole core could not only improve the charge transfer mobility, but also convert p-type materials into ambipolar type semiconductors (Figure 1H) (Zhang et al., 2018c; Zhang et al., 2019a).

## Sensors

A sensor is a type of detection device that can respond to external conditions and transform the stimulation into electrical signals or other required output information to meet the requirements of information transmission, storage, control, monitor, display, as well as recording (Charoenthai et al., 2011; Pattanatornchai et al., 2013; Xu et al., 2013; Lu et al., 2014).

Sensors are one of the key applications of conjugated conductive polymers. In most cases, sensors provide a signal as a response to the stimulation of environmental conditions such as light, gas, anions, and solvents. In 2020, Stewart et al. developed a volatile organic compound (VOC) gas sensor with highly sensitive and selective characteristics using p-conjugated polymer (P3HT)/solid-state ionic liquid (SSIL) blends. They determined that the strong chemical interaction between π-CP and SSIL adjusts the conductivity by applying an electric field (Stewart et al., 2020). The sensor will exhibit different conductivity and transmit signals to distinguish different substances under exposure with VOCs of different polarity. The results show that the sensor has better accuracy and stability. This work proved that conjugated conductive polymers have potential applications in the creation of more accurate, cheaper, and easier-to-fabricate sensing arrays.

In addition to gas, the conjugated polymers change colour under different temperatures which can be used for temperature monitoring, biosensors, building materials, and intelligent screens. Kamphan et al. introduced different lengths of side chains into a polydiacetylene (PDA) system, resulting in a series of PDA-based polymers. These polymers exhibited λ_max_ at approximately 640 nm, where λ_max_ shifted to approximately 585 nm when the temperature increased to 75°C, that is, a colour change from blue to purple was noticed (Figure 2A) (Kamphan et al., 2016). The colour change might be because the extended π-conjugation system results in an absorption peak at 640 nm, appearing blue, while the twisting of the polymer backbone as well as the changes in the degree of conjugation in the system result in a red colour. Recently, a series of PDA-based polymers with reversible colour change responses to temperature have been reported; these could be potentially used in various applications, including biomedicine, optical storage, sensing, building materials, and anti-counterfeiting.

Various anions exist in the environment. Although anions are generally beneficial for human health, an excessive amount is disadvantageous for human and animal health. Thus, the development of highly sensitive and selective sensors is important. These materials can also be found in conjugated conductive polymers. Recently, Zhang et al. developed a diketopyrrolo[3,4-c] pyrrole (DPP)-based polymer with NH units on the polymer backbone. The functional group NH reacts with the fluoride anions in the organic solvents, resulting in a colour change from red to blue, which is visible to the naked eye (Figure 2B). Moreover, these polymers not only detect the fluoride anion with high sensitivity and selectivity with a detection limit as low as 10–^8^ M, but also work as fluoride anion extractors (Zhang et al., 2018b). Very recently, some novel designed insulated conjugated bimetallopolymer for the sensor application were also developed ( Kaneko et al., 2020; Masai et al., 2020).

## Coating

Coating is a material that covers the surface of objects *via* different construction processes to form a solid film with firm adhesion, a certain strength, and continuity. This kind of material is widely used in building decoration, functional coating, intelligent control, and other daily-life applications.

Further development of high-performance coatings is necessary (typically functionality coating) as they have significantly improved daily life. Recently, Zhang and Zhou et al. introduced conjugated benzodifuranone dyes into an epoxy-polyamine-composited polymer system (Zeng et al., 2019). This coating exhibited a change from dark blue into yellow in response to a change in temperature, PH values, or after exposure to UV-light (Figure 2C). Subsequently, the same group investigated the mechanism and found that the colour change is ascribed to the dissociation of the hydrogen bonding (Zhang et al., 2019b). These types of coatings are potentially used in temperature or UV-light sensors.

Coating, on the top of the materials, worked as a protective functionality. In 2009, Yan et al. reported a neutral conjugated polymer with the ability to cathodically protect exposed alloy in a defect, which was the first report of cathodic protection of an all-organic coating based on a conjugated conductive polymer. In this experiment, SVET current density maps were generated to illustrate the ability of the reaction with the oxygen of the neutral conjugated polymer (Yan et al., 2009). The results showed that oxidation current centered on the defect and most of the reduction current distributed on the surface of the polymer after 2 h, which determined that the polymer is oxidised on the cathode instead of the metal electrode so as to better protect the cathode. In summary, this type of material can protect the cathode. Applications for conjugated conductive polymer coatings are currently being investigated.

## Conclusion and Future Prospects

In summary, a series of conjugated conductive polymers and their applications are reviewed. Regarding OSC-conjugated materials, novel chemical structures with broad optical absorption (typically absorbing light from the visible light to the NIR region), high charge mobility, and low LUMOs as non-fullerene acceptors are popular. Conjugated polymers with multi-fluorides, nitrogen, and sulfur atoms in the backbone are promising. High PCE and device stability are important to modify the OSCs. The chemical structures of conjugated polymers, typically the backbone, play a key role in the performance OFETs. To obtain high charge mobility semiconductors, the materials should be produced taking all factors into account, including a good planar backbone, strong aggregating and π–π stacking, and increased crystallinity. In addition, n-type and ambipolar type semiconductors are essential as the most successful electron mobility of OFETs is much smaller than the hole transfer mobility. Regarding sensors, highly selective and sensitive sensors still require further research and development of conjugated polymers. Different applications of sensors have different molecular design concepts. Traditional coatings act as a protective material, while several of them alter the colour of the surfaces. The development of conjugated polymer coatings with multiple functions is promising, including the coating combined as semiconductors and sensors. In the near futures, applications regarding to conjugated polymers are expected to be developed in the direction of interdisciplinary such as OFETs with sensitive of X-ray or light sensors, bio-sensors with functionality of cure disease and so on. In addition, the conjugated polymers used in the energy storage and light-emitting devices are also should be further developed.
